# An exploratory study of metabolomics in endogenous and cannabis-use-associated psychotic-like experiences in adolescence

**DOI:** 10.1038/s41398-024-03163-9

**Published:** 2024-11-07

**Authors:** Karoliina Kurkinen, Olli Kärkkäinen, Soili M. Lehto, Ilona Luoma, Siiri-Liisi Kraav, Petri Kivimäki, Sebastian Therman, Tommi Tolmunen

**Affiliations:** 1 Institute of Clinical Medicine, University of Eastern Finland, Yliopistonranta 1, FI-70210 Kuopio, Finland; 2 School of Pharmacy, University of Eastern Finland, Yliopistonranta 1, FI-70210 Kuopio, Finland; 3 Institute of Clinical Medicine, University of Oslo, P.O. Box 1171, Blindern, 0318 Oslo, Norway; 4 R&D Department, Division of Mental Health Services, Akershus University Hospital, PB 1000, 1478 Lørenskog, Norway; 5 Department of Psychiatry, Faculty of Medicine, University of Helsinki, Yliopistonkatu 3, 00014 Helsinki, Finland; 6 Department of Child Psychiatry, Kuopio University Hospital, Kaartokatu 9, Kuopio, Finland; 7 Department of Social Sciences, University of Eastern Finland, Yliopistonranta 1, 70210 Kuopio, Finland; 8 Mental Health Team, Finnish Institute for Health and Welfare, P.O. Box 30, FI-00271 Helsinki, Finland; 9 Kuopio University Hospital, Department of Adolescent Psychiatry, Kaartokatu 9, Kuopio, Finland

**Keywords:** Psychiatric disorders, Biomarkers, Pathogenesis

## Abstract

In adolescence, psychotic-like experiences (PLE) may indicate potential prodromal symptoms preceding the onset of psychosis. Metabolomic studies have shown promise in providing valuable insights into predicting psychosis with enhanced precision compared to conventional clinical features. This study investigated metabolomic alterations associated with PLE in 76 depressed adolescents aged 14–20 years. Serum concentrations of 92 metabolites were analyzed with liquid chromatography–mass spectrometry. PLE were assessed using the Youth Experiences and Health (YEAH) questionnaire. The associations between PLE symptom dimensions (delusions, paranoia, hallucinations, negative symptoms, thought disorder, and dissociation) and metabolite concentrations were analyzed in linear regression models adjusted for different covariates. The symptom dimensions consistently correlated with the metabolome in different models, except those adjusted for cannabis use. Specifically, the hallucination dimension was associated with 13 metabolites (acetoacetic acid, allantoin, asparagine, decanoylcarnitine, D-glucuronic acid, guanidinoacetic acid, hexanoylcarnitine, homogentisic acid, leucine, NAD^+^, octanoylcarnitine, trimethylamine-N-oxide, and valine) in the various linear models. However, when adjusting for cannabis use, eight metabolites were associated with hallucinations (adenine, AMP, cAMP, chenodeoxycholic acid, cholic acid, L-kynurenine, neopterin, and D-ribose-5-phosphate). The results suggest diverse mechanisms underlying PLE in adolescence; hallucinatory experiences may be linked to inflammatory functions, while cannabis use may engage an alternative metabolic pathway related to increased energy demand and ketogenesis in inducing PLE. The limited sample of individuals with depression restricts the generalizability of these findings. Future research should explore whether various experiences and related metabolomic changes jointly predict the onset of psychoses and related disorders.

## Introduction

Psychosis is a devastating condition, and there is growing interest in identifying biomarkers that can predict its occurrence during the prodromal stage, preceding actual onset [1, 2]. For instance, plasma proteomics have been found to predict psychosis with greater accuracy than clinical features [3]. Early diagnosis is particularly important for implementing interventions that result in better recovery from the first psychotic episode [4]. Based on earlier studies, prodromal, first-episode, and chronic stages of psychosis share similar alterations in lipid and glucose metabolism, which may thus form promising biomarkers [5, 6].

The physiological phenomena associated with psychotic-like experiences and prodromal psychotic symptoms have been extensively studied, yet a clear consensus remains to be established. For example, lysophosphatidylcholines, lipids found to be altered in the prodromal stages of psychosis, have been implicated in promoting inflammation, which is another system associated with a higher risk of psychosis in adolescence [6]. Additionally, cAMP signaling, important in the integration of information from neurotransmitter receptors (e.g., glutamatergic, dopaminergic, and GABAergic receptors), has been implicated in the pathophysiology of psychosis [7]. Recognizing the interconnectedness of various systems and their impact on each other’s functions may unveil layers of development of disorder within the nervous system and at the systemic level. Conversely, subtypes such as autoimmune-related psychosis have been proposed, suggesting diverse mechanisms underlying psychoses [8].

Alterations in the plasma lipidome in children have been observed to precede psychotic-like experiences (PLE) [9] and psychosis [6] in adolescence, and dysregulated lipid metabolites have been found to predict psychosis in young adults [10, 11]. Furthermore, psychotic experiences in early adulthood have been associated with disturbances in lipid metabolism [12]. In addition, altered lipid levels in red blood cell membranes have been associated with an increased risk of psychosis [13]. Specifically, a disturbed biosynthesis of unsaturated fatty acid pathway and altered triacylglycerol levels have been found in both serum and plasma samples in patients clinically at high risk of psychosis [10–12].

Other common findings related to the prodromal stages of psychosis are altered serum and plasma levels of phosphatidylcholines, lysophosphatidylcholines, and sphingomyelins [6, 10, 12]. In particular, alterations in phosphatidylcholines and lysophosphatidylcholines during childhood have been observed to precede the manifestation of PLE in adolescence [9]. Similarly, phospho- and sphingolipids have been found to be altered in first-episode psychosis (FEP) when compared to healthy controls [5]. Apolipoprotein E, an important protein in cholesterol metabolism, has been present at greater levels in adolescents undergoing persistent psychotic experiences compared to those whose experiences did not persist [14]. Findings in individuals with an interview-assessed clinical high risk of psychosis (CHR) include altered catecholamine dopamine and noradrenaline metabolite alterations in saliva samples [15]. Additionally, PLEs have been associated with changes in gene expression, observed as altered DNA methylation [16], as well as alterations in the proteome [14, 17] in children and adolescents when compared to healthy age-matched controls. To our knowledge, no metabolomic research, except for lipidomic studies, has been conducted in relation to PLE.

While metabolomic changes appear to be associated with the physiological process of psychosis, the chronicity of a disease and medications can also impact the metabolome [5, 18]. Therefore, the investigation of unmedicated patients at risk of or in the early stages of a disorder is crucial for a better understanding of the early disease etiology. In order to identify metabolomic changes linked to the initial stages of the psychotic process, we conducted an exploratory study to investigate the associations between PLEs and the metabolome in a cohort of 14–20-year-old, mainly unmedicated depressed psychiatric outpatients.

## Methods

### Study population

The present study formed part of the SMART (Systemic Metabolomic Alterations Related To different psychiatric disease categories in adolescent outpatients) project, which has recruited 14–20-year-old patients from the Adolescent Psychiatry Outpatient Clinic at Kuopio University Hospital. When the current study was performed, 445 patients had been interviewed using the clinician version of the Structured Clinical Interview for DSM-IV (SCID-IV) [19] as part of the SMART project, all of whom responded to the questionnaires. The first 76 enrolled individuals diagnosed with a depressive disorder were included in this cross-sectional baseline study. The Research Ethics Committee of the Kuopio University Hospital reviewed and approved the SMART project in 2017.

### Questionnaires and clinical assessments

The psychotic-like experiences of the patients were assessed with the novel Youth Experiences and Health (YEAH) questionnaire [20], which incorporates 39 items previously shown to be predictive of psychosis or correlated with concurrent CHR symptoms (Supplementary Table 7), reformulated to a six-point frequency scale (from *many times/day* to *more rarely or never*). The 21-item Beck Depression Inventory (BDI-1A) was used to assess the severity of depressive symptoms, such as alterations in cognition, feelings, and physical symptoms, on a scale from 0–63 [21]. Adverse childhood events were assessed with the Trauma and Distress Scale (TADS), with a total raw score ranging from 0 to 100 [22]. Quality of sleep was measured with the Pittsburgh Sleep Quality Index (PSQI) [23] and the severity of insomnia with the Insomnia Severity Index (ISI) [24]. Alcohol use was evaluated with the first three questions of the Alcohol Use Disorders Identification Test (AUDIT-C), scored from 0–12 [25]. The tobacco (scored 0–31) and cannabis (scored 0–39) use scales of the ASSIST 3.1 interview were used to assess tobacco and cannabis use during the previous three months [26]. Higher scores indicate greater disturbance on all the above scales. Diet quality was evaluated with an adjusted 16-item version of the Index of Diet Quality (IDQ), with higher scores indicating a healthier diet. The IDQ has been developed according to Nordic nutrition recommendations to depict diet quality and health-promoting aspects of the diet, and it has been validated in the Finnish population [27]. To assess the overall use of medications, a dichotomized variable was used. The use of any medications was considered as ongoing medication in the variable. In addition, antipsychotic medication and SSRI use were included as separate dichotomized variables in the analyses.

### Blood sampling

Blood samples were obtained between 7–10 am after 12 hours of fasting, rested for 30 min, and centrifuged at 2500 x g for 10 min. After preparation, serum samples were stored at -70 °C. Analysis was conducted in one batch after the sample collection had been completed. Blood samples were collected and stored by the Kuopio University Hospital (KUH) laboratory unit ISLAB.

### Targeted metabolomics analysis

Metabolomics analysis was carried out at the Institute of Molecular Medicine Finland in Helsinki, Finland. Targeted metabolomics analysis was performed with ultra-performance liquid chromatography coupled to mass spectrometry (UPLC-MS). Ten microliters of radioisotopically labeled internal standard was added to 100 µL of sample, and the resulting mixture was allowed to equilibrate, after which 400 µL of extraction solvent (1% formic acid in acetonitrile) was inserted into the mixture and the resulting supernatant was collected. The supernatant was divided between the wells of a 96-well plate and filtered on a Hamilton Robotics vacuum station (300–400 mbar for 2.5 minutes). Five microliters of preprocessed sample was inserted into an ACQUITY UPLC® system coupled to a Xevo® TQ-S triple quadrupole mass spectrometer (Waters Corporation, Milford, MA, USA). UPLC-MS was run in positive and negative polarities, with the polarity switching time being 20 ms for the separation and quantification of the metabolites. The multiple reaction monitoring (MRM) acquisition mode was used for the quantification. MassLynx 4.1 software was used for data collection, handling, and instrument control, and Target Lynx software for data processing.

The resulting 92 metabolite concentrations provided us a targeted summary of each patient’s metabolism. The metabolites included acylcarnitines, amino acids and their derivatives, bile acids, carbohydrates and carbohydrate conjugates, cholesterol and steroid metabolites, choline metabolites, citric acid cycle metabolites, enzyme cofactors, ethanol amines, methylation cycle metabolites, neurotransmitter metabolites, nucleobases, nucleosides, nucleotides, organic substances, and products of the urea cycle. The results reflect the homeostatic state of biological processes, such as functions of the immune system and the energy metabolism of cells.

### Statistical analysis

#### Factor analysis of PLEs

The larger questionnaire data set (*n* = 445) was used to estimate a confirmatory item factor model of the YEAH questionnaire responses, resulting in six factors: delusions, paranoia hallucinations, negative symptoms, thought disorder, and dissociative symptoms. Factor analysis was performed with Mplus 8.3 software [28] using the default settings with the WLSMV estimator and theta parameterization. The pairwise coverage among the items ranged from 97.7% to 98.6% on average, indicating a minimal impact of missing data assuming that it occurred randomly. The model demonstrated an acceptable fit: CFI. 974, RMSEA. 052, and SRMR. 057. Standardized factor loadings and response thresholds are detailed in Supplementary Table 2b, c, with item-wise categorization provided in Supplementary Table 2a. Factor scores for subsequent analyses were derived using the maximum *a posteriori* method.

#### Regression models

The associations between factor scores of the six YEAH PLE dimensions and the individual metabolites were estimated in linear regression models with a custom script in the R statistical software environment (v 4.3.1) [29] using R packages *stats* (v 3.6.2; R builtin) and *lm.beta* (v 1.7-2) [30]. The metabolite concentrations were standardized to z-scores before linear regression modeling. Background variables with possible effects on metabolism were used as covariates in the regression models, which were based on previous findings indicating relevance, as described in our previous article [31]. These included gender, age, BMI, IDQ, ongoing medications, depression chronicity, BDI, TADS [32], ISI, PSQI, tobacco smoking, cannabis use [33], and alcohol use (ASSIST and AUDIT-C). In addition to the unadjusted Model 1, five adjusted linear models were estimated with the covariates correlating with the six symptom dimensions (Supplementary Table 3). As there were multiple variables to consider, overfitting was avoided by dividing the variables into five models. Model 2 was adjusted for the participants’ lifestyle effects with BMI and IDQ scores, Model 3 with ASSIST Tobacco, and Model 4 with ASSIST Cannabis scores. Model 5 was adjusted for mental health variables, considering TADS and BDI scores, and Model 6 was adjusted for sleep quality and insomnia severity with ISI and PSQI scores. Each resulting regression beta coefficient was hierarchically clustered in a heat plot with the R packages *gplots* (v 3.1.3) [34] and *RColorBrewer* (v 1.1-3) [35]. As a *post hoc* analysis, we performed linear regression analyses predicting metabolite concentrations with cannabis use (Supplementary Table 4).

#### Sensitivity analyses

Considering the non-normal distribution of some of the metabolites (Supplementary Table 5), we performed a sensitivity analysis in which rank transformed metabolite concentrations were analyzed with the linear model predicting the hallucination symptom dimension. The comparison of resulting estimates and 95% confidence intervals of original and rank normalized data was illustrated in a dot-and-whisker plot generated using the R package *ggplot2* (v 3.4.4) [36].

#### Principal component analysis of metabolite concentrations

In addition to linear regression analyses with individual metabolites, a multivariate principal component analysis (PCA) of metabolite concentrations was computed with SIMCA (Version 17; Sartorius Stedim Data Analytics AB) for data reduction, and primary components were correlated with PLE factor scores. As metabolites tend to correlate with each other in targeted metabolomic analyses, multiple testing was used to adjust the *α* level [37]. The *α* level of 0.05 was divided by the number of PCA components explaining at least 95% of the variation in the data. Metabolomic associations with *p-*values of less than 05 and more than the *α* adjusted for multiple testing were considered as trends. Metabolites with over 10% missing cases were excluded from the analyses, except for cotinine, a molecule naturally absent in nicotine-naïve people.

## Results

### Demographic and clinical variables

The demographic and clinical characteristics of the sample and their associations with the six PLE factors (1. delusions, 2. paranoia, 3. hallucinations, 4. negative symptoms, 5. thought disorder, and 6. dissociation) are presented in Table 1. From this sample, 65.8% of the patients had recently had experiences of delusions, 96.1% paranoia, 78.9% hallucinations, 96.1% negative symptoms, 89.5% thought disorder and 94.7% dissociation at least occasionally. None of the characteristics were associated with all six factors. Delusions were associated with a higher BMI and lower IDQ, higher BDI, TADS, ISI, and PSQI scores, and chronicity of depression. Paranoia was associated with tobacco smoking, cannabis use and antipsychotic medication, as well as higher BDI and TADS scores. Hallucinations were associated with cannabis use and with higher BDI, TADS, ISI, and PSQI scores. Negative symptoms were associated with tobacco smoking, episodic MDD, and higher PSQI scores. Thought disorder was associated with cannabis use and higher BDI, ISI, and PSQI scores. Dissociation was only associated with higher BDI scores. We did not observe any of the factors to correlate with gender, age, alcohol consumption, or overall medication use (Table 1). Of the sample population, 32% were taking SSRIs, 17% antipsychotics, 5% agomelatine, tricyclic antidepressants or vortioxetine, 1% mood stabilizers, 8% mirtazapine, and 32% other medications, including melatonin, mini-pills, beta blockers, insulin or oxazepam. The 95% confidence intervals of the betas are additionally presented in Supplementary Table 1.

**Table 1:** Characteristics of the participants, including distributions of multivariate model covariates, with standardized linear regression coefficients in models predicting PLE dimensions.

Variable	Cohort		Delusions		Paranoia		Hallucinations		Negative symptoms		Thought disorder		Dissociation	
			*β*	*p*	*β*	*p*	*β*	*p*	*β*	*p*	*β*	*p*	*β*	*p*
Gender; Male *n* (%)	12	(16)	0.22	0.057	0.07	0.556	0.16	0.165	−0.15	0.188	−0.02	0.855	−0.02	0.839
Age, mean (SD)	16.4	(1.6)	0.06	0.624	−0.14	0.241	−0.08	0.476	−0.02	0.883	−0.04	0.756	0.19	0.097
Body mass index, mean (SD)	22.3	(5.7)	**0.29**	**0.012**	−0.01	0.943	0.09	0.427	0.03	0.772	0.09	0.454	0.22	0.062
Diet quality (IDQ), mean (SD)	25.6	(5.7)	**−0.27**	**0.020**	0.18	0.117	−0.10	0.403	0.12	0.319	−0.04	0.744	−0.17	0.147
Tobacco use (ASSIST), mean (SD)	6.5	(9)	−0.08	0.475	**0.24**	**0.037**	0.09	0.433	**0.29**	**0.012**	0.17	0.141	−0.02	0.866
Cannabis use (ASSIST), mean (SD)	2.3	(3.4)	0.08	0.134	**0.56**	**0.031**	**0.72**	**0.003**	0.36	0.185	**0.54**	**0.040**	0.47	0.075
Alcohol use (ASSIST), mean (SD)	8.1	(8.3)	−0.07	0.627	0.21	0.115	0.02	0.879	0.09	0.525	0.06	0.638	−0.18	0.166
Alcohol use (AUDIT-C), mean (SD)	2.7	(2.9)	−0.22	0.057	0.06	0.620	−0.13	0.277	0.22	0.054	−0.01	0.960	−0.08	0.507
Medication, *n* (%)	43	(56.6)	0.21	0.914	−0.08	0.608	−0.22	0.255	0.01	0.936	0.33	0.055	−0.08	0.588
SSRI, *n* (%)	24	(31.6)	0.11	0.508	−0.11	0.508	−0.20	0.306	0.02	0.926	0.31	0.079	−0.03	0.829
AP medication, *n* (%)	13	(17.1)	0.14	0.471	**−0.33**	**0.038**	0.15	0.935	0.13	0.402	0.24	0.151	0.03	0.831
Chronic depression, *n* (%)	43	(56.6)	**0.22**	**0.017**	−0.09	0.462	0.07	0.526	**−0.24**	**0.034**	−0.06	0.714	0.12	0.307
Depression (BDI), mean (SD)	30.2	(7.6)	**0.30**	**0.008**	**0.32**	**0.005**	**0.43**	**<0.001**	0.14	0.229	**0.43**	**<0.001**	**0.27**	**0.019**
Childhood adversity (TADS), mean (SD)	43.8	(18.6)	**0.27**	**0.017**	**0.34**	**0.003**	**0.32**	**0.007**	0.11	0.337	0.21	0.071	0.14	0.235
Insomnia (ISI), mean (SD)	12.6	(5.4)	**0.27**	**0.019**	0.15	0.208	**0.27**	**0.018**	0.20	0.081	**0.17**	**0.018**	0.19	0.097
Sleep quality (PSQI), mean (SD)	10.7	(3.9)	**0.27**	**0.020**	0.19	0.101	**0.33**	**0.004**	**0.29**	**0.010**	**0.33**	**0.004**	0.21	0.074

Values with *p* ≤ 0.05 are highlighted in bold. *AP* antipsychotic, *ASSIST* Alcohol, Smoking and Substance Involvement Screening Test, *AUDIT-C* Alcohol Use Disorder Identification Test, *BDI* Beck Depression Inventory, *IDQ* Index of Diet Quality subset, *ISI* Insomnia Severity Index, medication coded as having any of agomelatine, mirtazapine, SSRI, antipsychotic medication, or other medications, *β* standardized regression coefficient, *p* *p* value of linear regression model (statistical significance), *PSQI* Pittsburgh Sleep Quality Index, *SD* standard deviation, *SSRI* medication with only selective serotonin reuptake inhibitors, *TADS* Trauma and Distress Score.

### Linear regressions predicting YEAH factors with metabolite concentrations

**Fig. 1:**
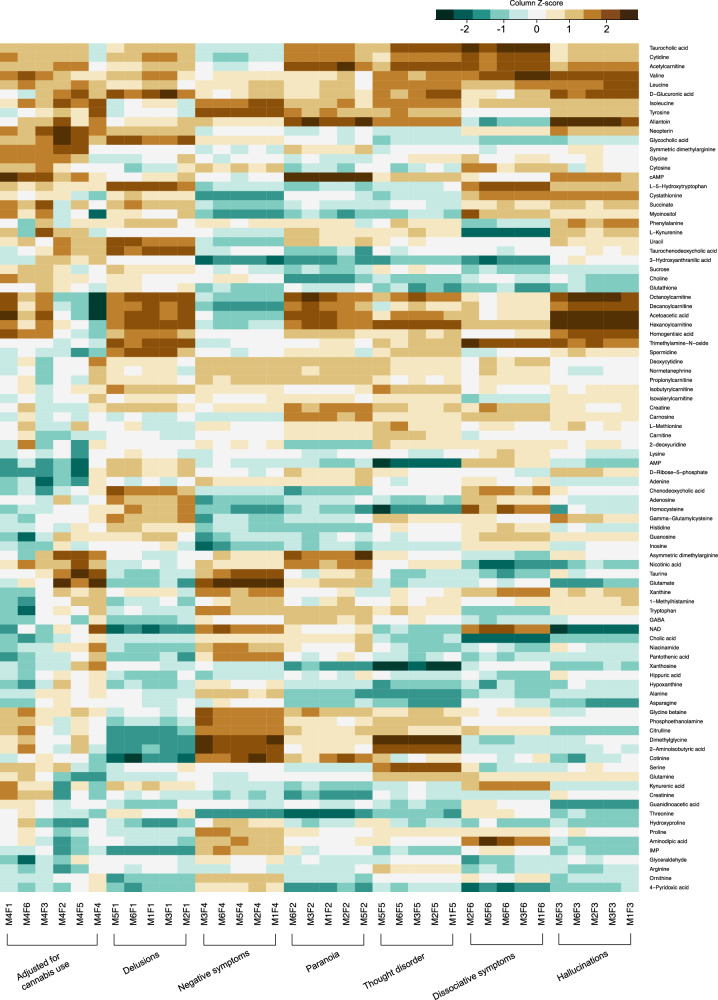
Overview of the linear regression results as a heatmap where *β*-values from the linear regression between each factor in each model and measured metabolites have been reordered and presented as *z*-scores with different colors. Both metabolites and models were hierarchically clustered. M model, F factor.

The results of linear regression analyses with six regression models and six PLE factors against 92 metabolites are displayed in Fig. 1. The results were similar for each factor in the various regression models, except for Model 4, which was adjusted for cannabis use, as can be seen on the left side of Fig. 1. The results are presented in greater detail (*β*, *p*, 95% CI) for each model and each factor in Supplementary Table 3. Factor 6, dissociation, had the smallest number of significant results in the linear regression, from one metabolite to three depending on the model, which is already expected by chance. In contrast, the number of significant metabolites varied between 8–13 in the six regression models with Factor 3, hallucinations.

**Fig. 2:**
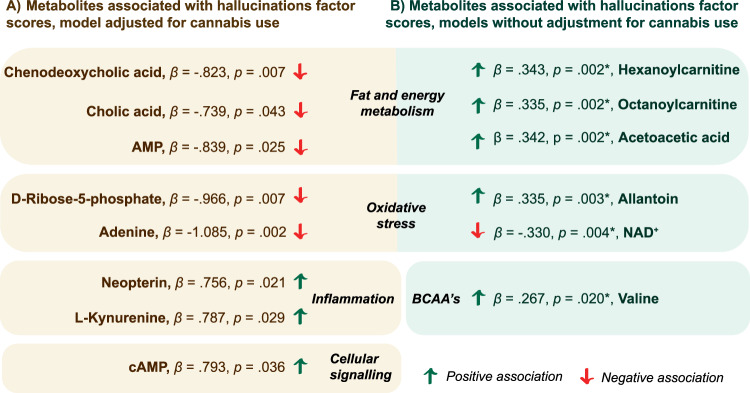
The statistically relevant findings from linear models of hallucination factor scores. Illustration of the results not observed in other models except for when cannabis use was adjusted for (**A**) and of the results observed in five models but not in M4 adjusted for cannabis use (**B**). AMP adenosine monophosphate, cAMP cyclic AMP, BCAA branched-chain amino acid. The associations that were the most significant are presented, if found in multiple models (*).

The metabolites associated with hallucinations (Factor 3) that were consistently significant throughout all models (except Model 4, adjusted for cannabis use) were acetoacetic acid, allantoin, hexanoylcarnitine, nicotinamine adenine dinucleotide (NAD^+^), valine, and octanoylcarnitine. On the other hand, the metabolites that were only significant in Model 4 when predicting hallucinations were adenine, adenosine monophosphate (AMP), cyclic adenosine monophosphate (cAMP), chenodeoxycholic acid, cholic acid, L-kynurenine, neopterin, and D-ribose-5-phosphate (Fig. 2). Other metabolites that were only found significant in some of the models when predicting hallucinations were asparagine, decanoylcarnitine, D-glucuronic acid, guanidinoacetic acid, homogentisic acid, leucine, and trimethylamine-N-oxide. The results for all six factors can be found in Supplementary Table 3.

### Sensitivity analyses

**Fig. 3:**
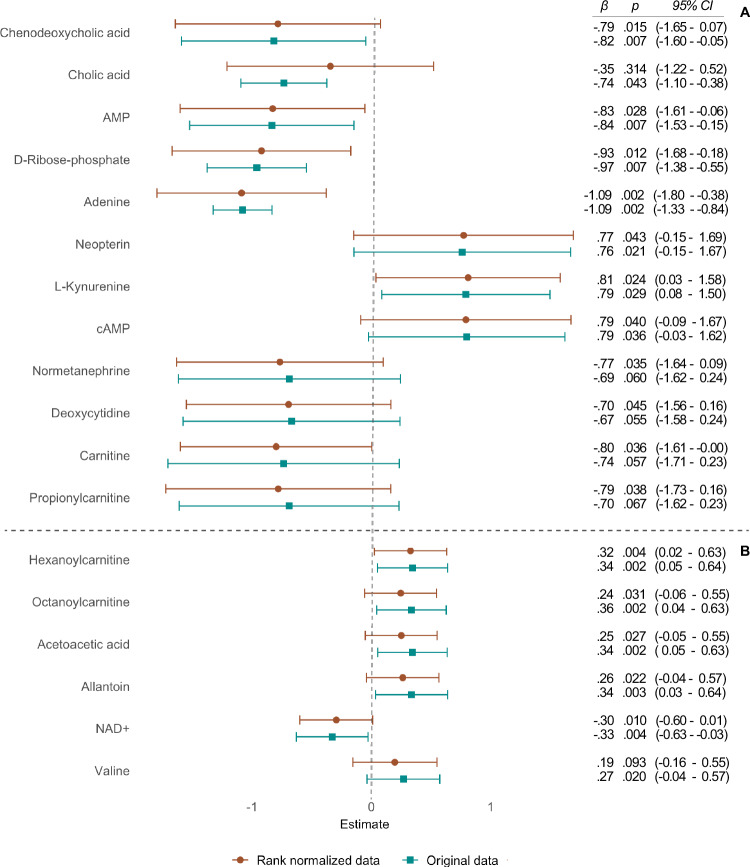
Dot-and-whisker plot of sensitivity analyses where estimates (standardized betas, *β*) are plotted with confidence intervals (95% CI) in the models using original and rank normalized data for the dimension hallucinations. **A** Models adjusted for cannabis use, **B** models without adjustment for cannabis use.

After implementing a distribution adjustment method (rank ordering) to even the data distribution and mitigate the effect of outliers, most metabolite associations remained consistent, although some variations were observed. Notably, cholic acid lost statistical significance in the model adjusted for cannabis use when employing rank normalization (Fig. 3A). However, chenodeoxycholic acid, AMP, D-ribose-5-phosphate, adenine, neopterin, L-kynurenine, and cAMP retained statistical significance after ranking. Some additional metabolites, on the other hand, reached statistical significance, including negative associations of carnitine, propionylcarnitine, normetanephrine, and deoxycytidine.

In models not adjusted for cannabis use, the ranking affected the results to some extent, as can be seen in Fig. 3B. Hexanoylcarnitine and NAD+ remained significant across all five models. Acetoacetic acid retained statistical significance in all models except those adjusted for BDI and TADS, while allantoin remained significant across all models except for one adjusted for PSQI and ISI. Octanoylcarnitine maintained statistical significance in models considering BMI and IDQ, as well as tobacco smoking, but not in models considering BDI and TADS or PSQI and ISI, nor the model without background variables. Valine lost statistical significance following rank normalization. All values from the sensitivity analysis in six models are presented in Supplementary Table 6.

### Principal component analysis

**Fig. 4:**
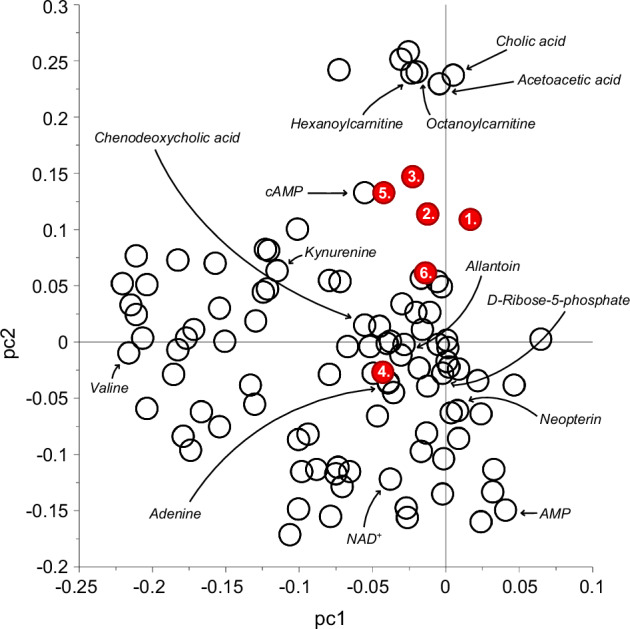
Factor means and individual metabolites plotted on the two first principal components of the 92 metabolites. Only the metabolites found associated with the factor hallucinations are named in this illustration. 1. Delusions, 2. Paranoia, 3. Hallucinations, 4. Negative symptoms, 5. Thought disorder, and 6. Dissociative symptoms. AMP adenosine monophosphate, cAMP cyclic AMP, NAD^+^ nicotinamide adenine dinucleotide.

In the PCA analyses, similar associations were observed between the YEAH PLE dimensions and metabolites as in linear regression models (Fig. 4). Some metabolites grouped very close to the hallucination dimension, for instance octanoyl- and hexanoylcarnitines, acetoacetic acid and cholic acid. Furthermore, the multiple-testing-adjusted α level was set to 0.0012, as 95% of the variation in the data was explained by 42 principal components. In this study, none of the results were below this level, and they should therefore be considered preliminary.

## Discussion

In this exploratory study, metabolomic alterations related to six psychotic-like experience dimensions were investigated. The highest number of associated metabolites were found to be linked to the frequency of hallucinations. Metabolites associated with non-cannabis-induced, endogenous hallucinatory experiences were related to inflammation, oxidative stress, cellular signaling, and fat and energy metabolism. The results also indicated that metabolites associated with ketogenesis and oxidative stress were linked to cannabis use or cannabis-induced PLEs.

When cannabis use was taken into account, we presume to have observed metabolomic alterations related to endogenous PLEs, whereas metabolomic alterations found in other models may reflect the direct effects of cannabis use or PLEs induced by cannabis use. An association between cannabis use, hallucinations, and schizophrenia-like psychoses has previously been observed [38], and cannabis use is a well-established risk factor for psychotic disorders [39]. This indicates that cannabis use may trigger an alternative pathophysiological pathway to psychotic symptoms in high-risk individuals. In fact, serum metabolomic profiles have been found in a preliminary report to differ between persons with schizophrenia, cannabis use disorder, or both [40].

### Metabolomic alterations related to hallucinations

#### Non-cannabis-related alterations

Chenodeoxycholic and cholic acids, as well as AMP, associated negatively with hallucinatory experiences in the model adjusted for cannabis use, suggesting that altered fat and energy metabolism is associated with non-cannabis-related hallucinatory experiences. However, cholic acid did not remain significant in the sensitivity analysis. These bile acids have a role in lipid absorption in the gut and cholesterol catabolism in the liver [41]. Levels of the same bile acids, namely cholic acid and chenodeoxycholic acid [42], have been found to be lower in schizophrenic patients. Furthermore, AMP is a nucleotide able to store energy in mitochondrial oxidative phosphorylation. Disturbances in oxidative phosphorylation have been suggested to play a role in schizophrenia pathology [43]. Similar decrease in AMP has been associated with CHR for psychosis when compared to healthy controls [11]. Moreover, carnitine and propionylcarnitine displayed negative associations with hallucinatory experiences in a model employing more robust normalization. Both compounds play roles in energy metabolism and the transportation of lipids into mitochondria [44].

Moreover, metabolites related to oxidative stress and inflammation were found altered in the model adjusted for cannabis use. The decrease in D-ribose-5-phosphate, an intermediate of the pentose-phosphate pathway (PPP) [45], and adenine, a purine catabolized to allantoin, may reflect an increased need for NADPH against oxidative stress [46, 47]. On the other hand, neopterin is an indicator of immune system activity [48] and the kynurenine pathway has been associated with inflammation [49], and both were associated positively with hallucinatory experiences. High serum neopterin has been found in persons with schizophrenia when compared to healthy controls, and antipsychotic medication has been found to significantly reduce the blood levels of neopterin [50]. On the contrary, decrease in kynurenine levels have been observed in CHR individuals [11]. A recent meta-analysis on schizophrenia revealed that the serum kynurenine/tryptophan ratio may be the only useful peripheral biomarker within the kynurenine pathway [51]. However, recent literature indicates the existence of a specific subtype of psychosis associated with autoimmune conditions [8]. In fact, alpha-2-macroglobuling, an inflammation-related protein, has been found to predict psychosis in the general population with PLE [3].

Finally, cAMP was positively correlated with hallucinations only when cannabis use was considered. cAMP signaling is part of the information integration from neurotransmitter receptors, and is found to be altered in patients with psychosis [7, 52]. Furthermore, disruptions in cAMP signaling in young adults with a clinical high risk of psychosis [52, 53] and increased cAMP levels in the olfactory neuronal precursor cells of persons with schizophrenia and bipolar disorder have been observed when compared to healthy controls [54].

#### Alterations related to cannabis use

Hexanoylcarnitine, octanoylcarnitine, and acetoacetic acid had positive trend-level associations with hallucinations in every model except for the one adjusted for cannabis use. Medium-chain acylcarnitines, such as hexanoyl-, octanoyl-, and decanoylcarnitines, support fat beta-oxidation, resulting in increased ketones in cells [55]. Acetoacetic acid is the stable form of its conjugate base acetoacetate, a ketone body. High levels of hexanoylcarnitine and octanoylcarnitine have been reported in first-episode psychosis and schizophrenia patients [18, 56]. Furthermore, antipsychotic treatment appears to alleviate high acylcarnitine levels [18]. Increased saliva acetoacetic acid levels have been detected before the onset of schizophrenia [15], and high blood levels have been observed in patients with schizophrenia [57]. Moreover, increased acetoacetic acid levels have been observed in CHR individuals, suggesting that its alterations may associate with PLE in this study [11].

However, it is possible that altered levels of acylcarnitines are caused by cannabis use per se. Hexanoylcarnitine had a direct positive association with cannabis use in this sample (Supplementary Table 4), and cannabis use has previously been suggested to alter carnitine synthesis pathways [58]. Cannabinoid effects on cellular energy metabolism have previously been reported [59]. However, these phenomena are not mutually exclusive. For instance, a dysregulated endocannabinoid system, which is closely connected to lipid metabolism, has been observed in FEP [38, 60], suggesting its potential involvement in the underlying pathophysiology of psychosis.

Alterations related to acylcarnitines and acetoacetic acid may suggest a pathway for prodromal psychotic symptomology induced by cannabis use via the mitochondrial production of ketone bodies for energy. Ketogenesis is an alternative energy source, especially in the liver and astrocytes [61]. The type-1 cannabinoid (CB_1_) receptor has been found to modulate astrocytic ketogenesis, and in a rat model, cannabinoids have been found to stimulate the production of ketone bodies, such as acetoacetic acid [61]. A ketogenic diet has also been studied as a possible augmentation treatment for schizophrenia [62].

Furthermore, the purine metabolite allantoin had a positive, and the oxidant NAD^+^ a negative trend-level association with the hallucinations dimension in all the models except for the one adjusted for cannabis use. Allantoin is generated from uric acid when reactive oxidative species (ROS) are present [63], and NAD^+^ can inhibit the production of reactive oxygen species in cells [64]. Furthermore, increased allantoin and decreased NAD^+^ have been reported in patients with schizophrenia, in line with our results [63, 65]. An increase in ROS while lacking antioxidants results in an increase in lipid peroxidation [66], while the beta-oxidation of lipids, discussed above, increases the formation of ROS in various CNS disorders [67]. In this study, molecules related to beta-oxidation and oxidative stress were coincidentally found to be altered.

Finally, the branched-chain amino acid (BCAA) valine displayed a positive trend-level association with the hallucination symptom dimension in all models except when cannabis use was considered, but this association did not remain significant after rank normalization. High levels of valine have been found in the plasma of unmedicated patients with schizophrenia [68]. On the contrary, low valine levels have been reported in first-episode psychotic patients [69]. However, a positive association between cannabis use and serum valine levels has also been reported [58], suggesting that high valine levels could be associated with cannabis use.

### Strengths and limitations

This study used a novel approach of investigating potential prodromal psychotic symptoms from a symptom dimension perspective. The patients were young, and our findings for some of them may therefore reflect metabolomic changes at a rather early stage before any psychosis onset. However, the study did not include follow-up data to confirm the prodromal nature of the psychotic-like experiences, which is an important limitation to be addressed in the following research. The sample was small, especially the subset reporting any cannabis use (*n* = 15, 20%), increasing type II bias in the results and highlighting that these results should be considered preliminary. Another limitation was that the study did not include a healthy control group. PLE has been suggested to be limited as they may not predict only psychosis but onset of a variety of psychiatric conditions [70], however, some dimensions have been found to more accurately predict psychotic conditions than others [71]. The choice of transformation method for metabolomics data is important, and in this study, more robust rank ordering was used as a sensitivity analysis in addition to the original data. Ranking changes the nature of the data, but the results were relatively similar in this study (Fig. 3). Other factors (e.g., perinatal complications) have also been associated with psychotic symptoms, which could not be controlled for in this study. However, several covariates, such as childhood adversity, either considered or potentially considered in this study, are likely to induce psychotic-like experiences. Therefore, including them as covariates in the analyses may be overly conservative. Behavioral changes resulting from either cannabis use or PLEs may also account for some of the observed alterations, although they were considered in the analyses (e.g., diet and sleep quality). Finally, females were overrepresented in this sample, reflecting the natural incidence of depression and a higher tendency to seek treatment and take part in studies among females. In future metabolomic studies considering psychosis-related phenomena, it will be important to consider cannabis use by patients. In addition, future analyses could include broader lipidomic assays, considering that numerous studies investigating psychotic-like experiences or prodromal stages of psychosis have reported alterations in lipids.

### Conclusions

In this exploratory study, we detected metabolomic alterations related to six different PLE dimensions. The degree to which these dimensions were associated with the peripheral markers varied, and we observed cannabis use to have an impact on the associations. Based on these preliminary results, we hypothesize that PLEs develop via various pathophysiological mechanisms, one being inflammation. Cannabis use, on the other hand, was associated with hallucinatory experiences via increased energy demand and ketogenesis. In the future, the prevalence of psychotic disorders later in life could be examined in the light of endogenous and cannabis-use-related prodromal metabolomic alterations, following participants via comprehensive national registries to determine how well the observed metabolomic alterations predict psychotic episodes or disorder onset later in life, both as such and together with clinical indicators.

## Supplementary information

Summary of supplementary material

Supplementary Tables 1-6

Supplementary Table 7

## Data Availability

The data supporting the findings of this study are available upon request from the corresponding author, KK. However, due to the sensitive nature of the information and to protect the privacy of the research participants, the data are not publicly accessible. The study’s ethical approval and the consent terms agreed upon by the participants specifically prohibit public sharing of the data, even in anonymized form.
