# Supplementary material for: An exploratory study of metabolomics in endogenous and cannabis-use-associated psychotic-like experiences in adolescence
Source: Transl Psychiatry. 2024 Nov 7;14:466. doi: 10.1038/s41398-024-03163-9 (PMC11543670; doi:10.1038/s41398-024-03163-9)
Supplement: Supplementary file 1 — Summary of supplementary material [file 41398_2024_3163_MOESM1_ESM.pdf]

Summary of supplementary materials

Supplementary Table 1: Presents additional values, including confidence intervals, related to cohort background characteristics.

Supplementary Table 2a: Shows YEAH factor loadings sorted by each factor, with item text provided in both Finnish and English.

Supplementary Tables 2c-b: Describes the full factor model, including standardized YEAH item factor loadings, thresholds, and factor correlations.

Supplementary Table 3: Presents linear models across six factors, with standardized beta, p-value, and confidence intervals. Various adjustment models are included based on lifestyle, mental health, and sleep quality variables.

Supplementary Table 4: Presents a post hoc analysis between history of cannabis use and the metabolites, exploring direct associations and potential explanations for the main findings identified in the study.

Supplementary Table 5: Provides basic statistical characteristics of metabolites for evaluating linearity of their distributions.

Supplementary Table 6: Presents linear models of rank-transformed metabolite concentrations in the hallucinations YEAH scale dimension as a sensitivity analysis.

Supplementary Table 7: Includes the original Youth Experiences and Health (YEAH) questionnaire, with validation information pending publication.
