# Supplementary material for: An exploratory study of metabolomics in endogenous and cannabis-use-associated psychotic-like experiences in adolescence
Source: Transl Psychiatry. 2024 Nov 7;14:466. doi: 10.1038/s41398-024-03163-9 (PMC11543670; doi:10.1038/s41398-024-03163-9)
Supplement: Supplementary file 3 — Supplementary Table 7 [file 41398_2024_3163_MOESM3_ESM.docx]

**YEAH Questionnaire, version 1 (2017)**

**Please select the response alternative that best describes your recent experience (about the last 3 months).**

|  | | **Many times/day** | **Daily** | **A few times/week** | **Weekly** | **Monthly** | **More rarely or never** |
| --- | --- | --- | --- | --- | --- | --- | --- |
| 1 | I seem to live through events exactly  as they happened before (déjà vu). |  |  |  |  |  |  |
| 2 | I'm not interested in other people. |  |  |  |  |  |  |
| 3 | I have had experiences with telepathy,  psychic forces, or fortune telling. |  |  |  |  |  |  |
| 4 | I have difficulty concentrating on anything,  for example on listening or reading. |  |  |  |  |  |  |
| 5 | I hear things that other people can't hear  like voices of people whispering or talking. |  |  |  |  |  |  |
| 6 | Others think I'm odd. |  |  |  |  |  |  |
| 7 | I feel that other people are watching me or  talking about me. |  |  |  |  |  |  |
| 8 | My life feels unreal or stage-managed. |  |  |  |  |  |  |
| 9 | I mistake shadows for people or noises for voices. |  |  |  |  |  |  |
| 10 | I'm not really interested in anything. |  |  |  |  |  |  |
| 11 | I'm unsure whether something I experience  is real or imaginary. |  |  |  |  |  |  |
| 12 | I have difficulties coping with my  usual activities or tasks. |  |  |  |  |  |  |
| 13 | I'm disturbed by the feeling that others  can read my thoughts. |  |  |  |  |  |  |
| 14 | I am too tired to do anything. |  |  |  |  |  |  |
| 15 | My thoughts are so strong that  I can almost hear them. |  |  |  |  |  |  |
| 16 | It feels like something strange or inexplicable is  taking place in myself or in my environment. |  |  |  |  |  |  |
| 17 | I feel strongly that I am (or will be)  an unusually important person. |  |  |  |  |  |  |
| 18 | I'm easily distracted by sounds around me. |  |  |  |  |  |  |
| 19 | I see things or people that others cannot see. |  |  |  |  |  |  |
| 20 | It feels like there's something  badly wrong with my body. |  |  |  |  |  |  |
| 21 | I have the sense that some person or force is  around me, even though I can't see anyone. |  |  |  |  |  |  |
| 22 | I hear unusual sounds like banging, clicking,  hissing, or ringing. |  |  |  |  |  |  |
| 23 | I feel that others have it in for me. |  |  |  |  |  |  |
| 24 | I feel that some person or force takes away  my thoughts, interferes with my thinking, or  puts thoughts in my head. |  |  |  |  |  |  |
| 25 | I hold beliefs that other people would find  impossible or really weird. |  |  |  |  |  |  |
| 26 | When something sad happens, I don't really feel sadness, or when something joyful happens,  I don't really feel happy. |  |  |  |  |  |  |
| 27 | My thinking feels worryingly confused or disturbed. |  |  |  |  |  |  |
| 28 | I try to avoid the company of other people. |  |  |  |  |  |  |
| 29 | I feel like I'm under the control of  some power other than myself. |  |  |  |  |  |  |
| 30 | I feel that I don’t exist or that I'm dead. |  |  |  |  |  |  |
| 31 | I feel like I'm being followed or spied upon. |  |  |  |  |  |  |
| 32 | Many thoughts in my head are  competing for my attention. |  |  |  |  |  |  |
| 33 | When I look at a person, or look at myself in a mirror,  I see the face change right before my eyes. |  |  |  |  |  |  |
| 34 | I have difficulties in carrying out ordinary activities (e.g., washing myself, dressing, biking, housework, going to the store). |  |  |  |  |  |  |
| 35 | I smell or taste unpleasant things that  other people can’t smell or taste. |  |  |  |  |  |  |
| 36 | I feel that someone has bad intentions towards me. |  |  |  |  |  |  |
| 37 | I notice special meanings directed at me,  for example in advertisements, shop windows,  or in the way things are arranged. |  |  |  |  |  |  |
| 38 | I have trouble controlling my speech,  behaviour, or facial expressions. |  |  |  |  |  |  |
| 39 | I don't want to be in school or at work. |  |  |  |  |  |  |

Thank you for responding!
